# Dynamic Neuromuscular Control of the Lower Limbs in Response to Unexpected Single-Planar versus Multi-Planar Support Perturbations in Young, Active Adults

**DOI:** 10.1371/journal.pone.0133147

**Published:** 2015-07-29

**Authors:** Bart Malfait, Filip Staes, Aijse de Vries, Annemie Smeets, Malcolm Hawken, Mark A. Robinson, Jos Vanrenterghem, Sabine Verschueren

**Affiliations:** 1 Musculoskeletal Rehabilitation Research Group, Department of Rehabilitation Sciences and Physiotherapy, Faculty of Kinesiology and Rehabilitation Sciences, KU Leuven, Leuven, Belgium; 2 Research Institute for Sport and Exercise Sciences, Faculty of Science, Liverpool John Moores University, Liverpool, United Kingdom; University of Rome Foro Italico, ITALY

## Abstract

**Purpose:**

An anterior cruciate ligament (ACL) injury involves a multi-planar injury mechanism. Nevertheless, unexpected multi-planar perturbations have not been used to screen athletes in the context of ACL injury prevention yet could reveal those more at risk. The objective of this study was to compare neuromuscular responses to multi-planar (MPP) and single-planar perturbations (SPP) during a stepping-down task. These results might serve as a basis for future implementation of external perturbations in ACL injury screening programs.

**Methods:**

Thirteen young adults performed a single leg stepping-down task in eight conditions (four MPP and four SPP with a specified amplitude and velocity). The amplitudes of vastus lateralis (VL), vastus medialis (VM), hamstrings lateralis (HL), hamstrings medialis (HM) EMG activity, medio-lateral and anterior-posterior centre of mass (COM) displacements, the peak knee flexion and abduction angles were compared between conditions using an one-way ANOVA. Number of stepping responses were monitored during all conditions.

**Results:**

Significantly greater muscle activity levels were found in response to the more challenging MPP and SPP compared to the less challenging conditions (p < 0.05). No differences in neuromuscular activity were found between the MPP conditions and their equivalents in the SPP. Eighteen stepping responses were monitored in the SPP versus nine in the MPP indicating that the overall neuromuscular control was even more challenged during the SPP which was supported by greater COM displacements in the SPP.

**Conclusion:**

The more intense MPP and SPP evoked different neuromuscular responses resulting in greater muscle activity levels compared to small perturbations. Based on the results of COM displacements and based on the amount of stepping responses, dynamic neuromuscular control of the knee joint appeared less challenged during the MPP. Therefore, future work should investigate extensively if other neuromuscular differences (i.e. co-activation patterns and kinetics) exist between MPP and SPP. In addition, future work should examine the influence on the neuromuscular control of the magnitude of the perturbations and the magnitude of stepping height and stepping distance.

## Introduction

Anterior cruciate ligament (ACL) injuries are very common during sports activities in the young, active population (16–40 years); affecting approximately 250,000 people per year in the US [1]. Approximately 70–84% of all ACL injuries involve a non-contact injury mechanism [2]. Non-contact ACL injuries occur mostly in a multi-planar way during high-velocity deceleration, pivoting or landing tasks [3] that often lead to high external knee joint loads. During the performance of sports activities, ground reaction forces can exceed 5–6 times the athlete’s bodyweight [4]. These high external knee joint loads have to be counteracted by adequate neuromuscular control strategies to protect the joint against excessive tibio-femoral movements and thereby prevent joint damage.

Dynamic knee stability is a complex interaction between static and dynamic restraints whereby neuromuscular control relies on an interaction of the visual, vestibular and somatosensory system [5]. Maintaining dynamic knee joint stability during sports activities requires a combination of pre-programmed muscle activity (feed-forward processes) as well as reflex-mediated muscle activity (feedback processes) [5]. Afferent somatosensory information is essential in these motor control processes as different types of mechanoreceptors in capsulo-ligamentous structures continuously feed information into the central system [6]. While previous work has shown that there is a relation between an ACL injury and dynamic knee joint stability [7,8], the causality between an ACL injury and an altered neuromuscular control remains unclear. Altered neuromuscular control around the knee joint might increase the stress on the ACL as several cadaveric [9,10] and in-vivo studies [11] have shown that quadriceps contraction induces tension and strain to the ACL, while an ACL injury might also lead to an altered neuromuscular control. To our knowledge, only few prospective studies have investigated the relation between an altered neuromuscular control and the ACL-injury risk. Zebis et al. [12] showed that a large difference in muscular activity (amplitude) between the vastus lateralis (VL) and hamstring medialis (HM) during the preparatory phase (10 ms before initial contact) of a side cutting manoeuver might have a predictive value for ACL injury risk determination.

The main goal of screening for ACL injury risk is to prevent ACL injuries by enabling clinicians to identify those at risk by objectively examining an athlete’s movement patterns and the control of the external moments acting on the lower limbs and developing neuromuscular training programs with the intention to improve at-risk movement patterns [13]. Different screening tools have been developed to assess the movement patterns of athletes during specific tests which try to mimic dynamic, injury-mechanism related movements and appropriate joint loading. In this respect, side-cutting manoeuvers [12], drop vertical jumps (DVJ) [4], single leg DVJ [14] and side-stepping tasks [15] have all been used previously to determine biomechanical and/or neuromuscular landing patterns that might have a “predictive” value for identifying those at risk or that might be related to these predictive parameters. They all have advantages (e.g. easy to use, applicable in clinical setting) and disadvantages (e.g. lack of standardization, single-planar) [16]. Remarkably each task requires slightly different dynamic neuromuscular responses and it is not well established which may provide better insight. Nevertheless, most of these screening tasks involve planned movements in which the environment does not change during the performance of the movement. In contrast, ACL injuries mostly occur during sudden unexpected, dynamic, multi-planar movements in which the environment does change (i.e. opponent who is defending, athlete tries to catch the ball, etc.) [17]. These unanticipated tasks provide a more realistic stimulus and consequently lead to greater joint loading [18]. Additionally, unpredictable changes in the environment are risk factors for lower limb injuries [19,20] because they influence the neuromuscular control of the knee joint by altering the afferent input in the complex central interactions [21].

Unexpected external perturbations provide a way of generating unpredictable changes to the environment that can be linked to neuromuscular activity patterns during real sports situations [22]. It is well known that the quadriceps and hamstrings muscle groups do contribute to the dynamic knee joint stability during sports activities [23]. However, unexpected events may influence and challenge neuromuscular control more than pre-planned movements [24]. Additionally, multiple studies [3,25] have shown that the “dynamic valgus” ACL injury mechanism is multi-planar and consists of hip adduction, knee internal rotation, knee abduction and foot pronation [26]. Therefore, the implementation of unexpected, multi-planar perturbations in screening programs might elicit an observable difference in neuromuscular control that allows us to distinguish those at risk from those that are not.

Only limited studies investigated neuromuscular activation patterns in different types of perturbations (either several magnitudes or different planes). Previous research indicated that no differences were found in magnitudes of the muscle activity of the hamstring lateralis (HL) and the rectus femoris when comparing single-planar rotational with single-planar translational perturbations [27]. Additionally, Horak et al. [28] and Szturm et al. [29] showed that the magnitude of muscle activity was scaled towards the amplitude, velocity and acceleration of the single-planar platform perturbations resulting in higher muscle activity levels in more challenging perturbation conditions. However, to our knowledge, neuromuscular responses to multi-planar perturbations have not been investigated yet and the differences in neuromuscular control compared to single-planar perturbations remain unknown. Studying the differences in responses to multi-planar versus single-planar perturbations is important taking into account that the development and implementation of multi-planar perturbations is definitely more costly as highly advanced equipment is needed while single planar perturbations may well suffice to challenge neuromuscular control strategies.

Therefore, our purpose was to address the importance of the implementation of an unexpected perturbation which perturbs the dynamic neuromuscular homeostasis in a similar way that the athletes’ neuromuscular control is challenged in real sports situations without compromising the ability to perform the screening task. The specific aim of this exploratory study was to compare the neuromuscular responses to multi-planar versus single-planar perturbations during a stepping down task. We hypothesized that multi-planar perturbations result in greater activity levels of the quadriceps and hamstrings compared to their equivalent single-planar perturbations. Additionally, greater muscular activity levels of the quadriceps and hamstrings were hypothesized during perturbations that are characterized by greater platform amplitudes, larger velocities and larger accelerations. These results might serve as a basis for future implementation of perturbations in screening programs.

## Materials and Methods

### Participants

**Fig 1:**
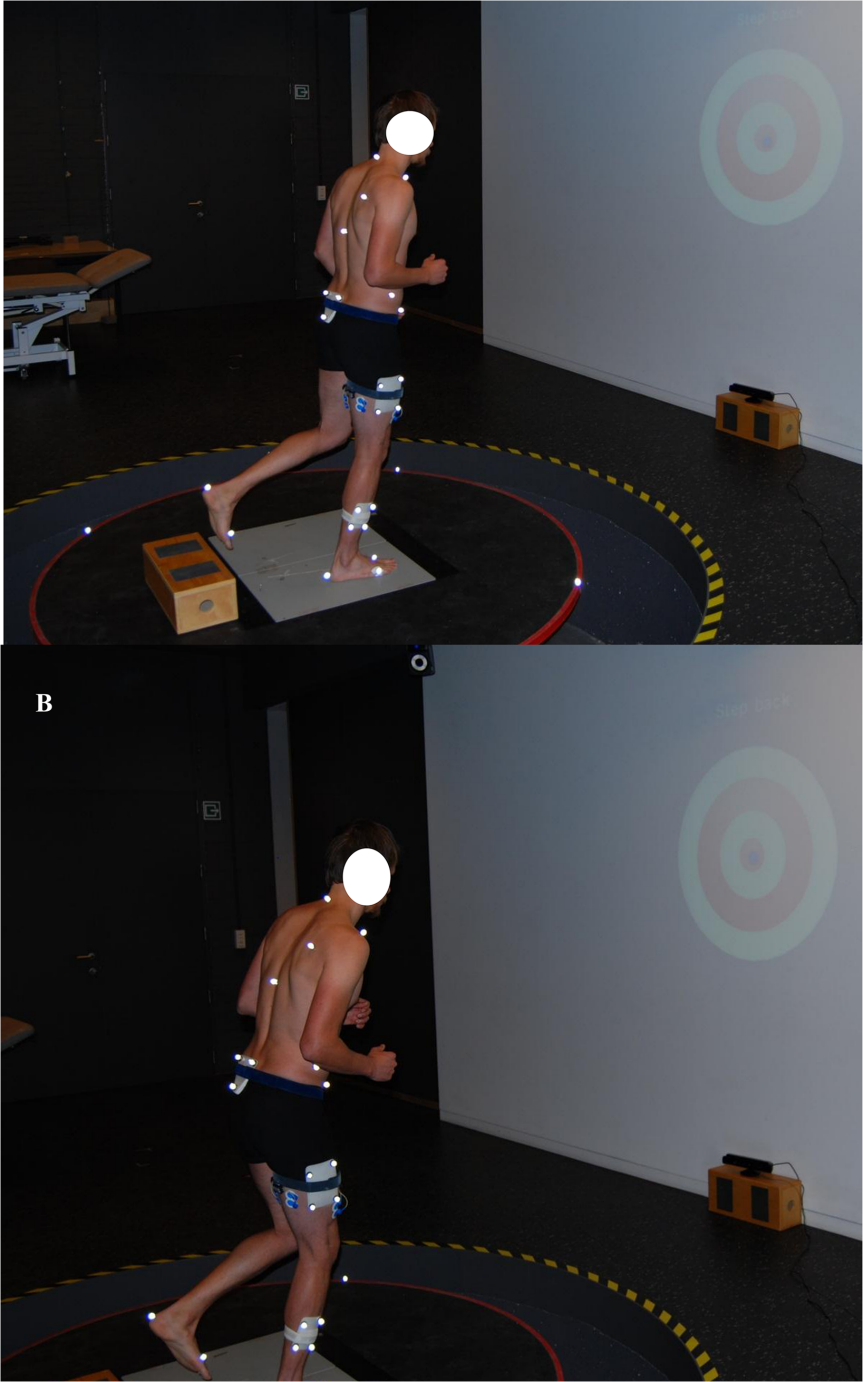
Visualisation of single-leg stepping down-task. A: Participant who performed the stepping down task with a more erect knee joint. B: Participant who performed the stepping down task in a more flexed pattern (more knee and hip joint flexion).

Thirteen recreationally active young adults (six males and seven females) volunteered in this study (23.2 ± 2.6 yrs, 1.7 ± 0.07 m, 64.2 ± 8.3 kg). All participants had no history of knee injury and were injury free for six months prior to data collection. Before participating in the study, all subjects read and signed the informed consent form. This study conformed to the principles of the declaration Helsinki (1964), was approved by the local ethics committee of the Biomedical Sciences, KU Leuven, Belgium and registered with reference number S56105. The individual in Fig 1 in this manuscript has given written informed consent (as outlined in PLOS consent form) to publish these case details.

### Design

All participants performed a standardized warm-up program consisting of two series of ten squats and ten vertical jumps [30]. During the “stepping-down task”, the participants were instructed to step off a box 0.1 m high and to land on their dominant leg on a force plate that was embedded within a six degrees-of-freedom moveable platform (Computer Assisted Rehabilitation Environment (CAREN) platform, Motek Medical, Amsterdam, The Netherlands) and to stand on this leg for five seconds (oral cue from the test leader) (Fig 1).All participants were barefooted. The participants were able to see the position of their foot via a real-time feedback application projecting the position of the stepping foot (virtual blue dot) onto a screen (in front of the subject). The participants had to hit the target (virtual red target) that was also projected on the screen. This virtual feedback system ensured that participants placed their foot on the same spot on the force plate during all trials and conditions. A metronome (66 beats/minute) was used so the participants performed the stepping task at a consistent pace.

The participants were told in advance that platform perturbations may occur. When 70% of the participants’ body weight was reached on the force platform during the stepping task, an unexpected perturbation would occur in one out of four stepping trials in a random order. Four small, medium, large and mixed multi-planar perturbations (MPP-S, MPP-M, MPP-L and MPP-X, respectively) and four single-planar perturbations (SPP-S, SPP-M, SPP-L and SPP-X, respectively) had been developed with specific amplitude, velocity and peak acceleration characteristics (Table 1). The mixed conditions consisted of the amplitude of the medium condition and the velocity of the large condition (Table 1), so enabling us to distinguish the amplitude effect versus the velocity effect. In total, all participants completed eight perturbation conditions that were presented in a random order using a Latin-square design. Each condition then consisted of twenty trials in which five perturbation trials were randomly interspersed with 15 non-perturbed trials. A one-minute rest period between consecutive conditions was permitted to avoid fatigue.

**Table 1:** Platform characteristics during the eight different conditions.

	Small	Medium	Large	Mixed
**MPP**	**MPP-S**	**MPP-M**	**MPP-L**	**MPP-X**
A-P amplitude (m)	0.021	0.041	0.062	0.041
M-L amplitude (m)	0.011	0.028	0.047	0.028
Rotation A-P-axis (°)	2.05	2.49	2.39	2.49
Rotation Vertical axis (°)	14.42	17.57	21.37	17.57
Average Velocity (m/s)	0.022	0.042	0.065	0.058
Peak Acceleration (m/s²)	0.397	0.575	0.792	1.380
**SPP**	**SPP-S**	**SPP-M**	**SPP-L**	**SPP-X**
M-L amplitude (m)	0.021	0.042	0.062	0.042
Average Velocity (m/sec)	0.023	0.046	0.069	0.062
Peak Acceleration (m/s²)	0.323	0.621	0.847	1.810

The multi-planar perturbations (MPP) were developed based on the literature [3,10,25] which describes an ACL injury mechanism resulting in a perturbation consisting of a posterior and lateral translation (inducing knee abduction) combined with a rotation around the anterior-posterior axis (inducing a foot pronation) and a rotation around the vertical axis (inducing external rotation). The single-planar perturbations (SPP) were purely translations in the lateral direction inducing knee abduction. Both MPP and SPP were programmed in D-flow (D-flow, Motek Medical, Amsterdam, The Netherlands) based on a sine-wave input [31]. Lees et al. [32] showed that the performance of the CAREN System is highly reliable for consistently reproducing movement patterns of sine wave or ramped perturbations.

### Data collection

Each participant had 44 spherical reflective markers positioned according to the eight segment ‘Liverpool John Moores University’ model (LJMU model) including feet, upper and lower legs, pelvis and trunk [30]. Segmental coordinate systems were defined as reported previously [33,34] using separate trials for anatomical calibration [35] and for calculating functional hip joint centres [36] and functional knee joint axes [34,37]. All modelling and analyses were undertaken in Visual 3D (v.4.83, C-MOTION, Germantown, MD, USA) using geometric volumes to represent segments based on cadaver segmental data [38]. Previous work of Malfait et al. [30] and Fauth et al. [39] showed that both lower limb kinematics and electromyographic measurements of the quadriceps and hamstrings muscle groups were highly reliable during dynamic tasks. The peak knee abduction angle during the performance of a drop vertical jump showed an inter-trial variability of 1.7° [30]. Fauth et al. [39] suggested that the EMG measurements of the quadriceps and hamstrings during dynamic activities are highly reliable as all intra-class coefficients were higher than 0.80.

A wireless EMG system (AURION, Italy) was used to record the muscle activity of the vastus lateralis (VL), vastus medialis (VM), hamstring lateralis (HL) and hamstring medialis (HM) using surface electrodes which were positioned according to the SENIAM guidelines. All electrode locations were shaved and gently cleaned with 70% isopropyl alcohol to reduce skin impedance. Silver-silver chloride, pre-gelled bipolar surface EMG electrodes (Ambu Blue Sensor, Ballerup, Danmark) were placed over the muscle belly and aligned with the longitudinal axis of the muscle, with a center-to-center distance of 0.02 m.

The stepping down tasks were registered by a 0.8 x 0.3 m AMTI (Watertown, MA, USA) force plate that was embedded in a 6 degrees-of-freedom hydraulic moveable platform. Force plate and EMG data were sampled at 1000 Hz. Three-dimensional kinematic data were simultaneously (time synchronized) recorded with the force and EMG data in Nexus (VICON, Oxford Metrics, UK) using 6 MX-T20 optoelectronic cameras (VICON, Oxford Metrics, UK) sampling at 100 Hz.

### Data analysis

**Fig 2:**
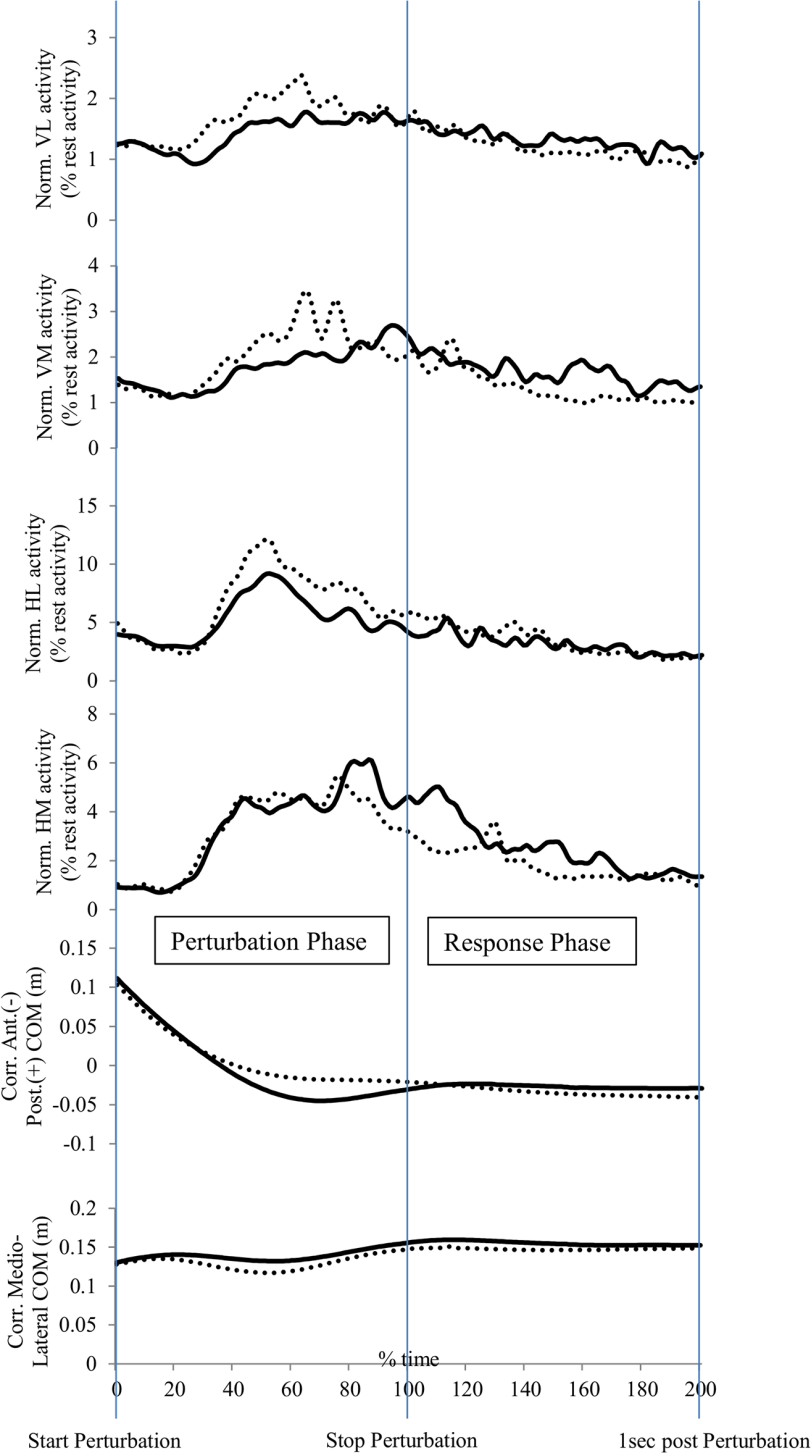
Data of all 13 participants to illustrate the different time periods. The straight line represents the average data for the large multi-planar perturbation (MMP-L) and the dotted line represents the average data for the large single-planar perturbation (SPP-L).

Whole-body kinematics were collected and processed in accordance with literature convention, however only the dominant leg was analysed and this was defined as the preferred leg for kicking a ball [40]. Marker trajectories and forces were filtered using a 4^th^ order low pass Butterworth filter with a cut-off frequency of 20 Hz [41]. Initial contact events were created when the vertical force crossed a 20 N threshold. All raw EMG signals were high pass filtered at a cut-off frequency of 10 Hz [42]. The rectified EMG signals were also filtered with a 4^th^ order zero-lag low pass Butterworth filter at a cut-off frequency of 10 Hz. The EMG signal amplitudes were subsequently normalized to the root mean square amplitude of the respective muscle activity during the first trial in which no perturbation occurred [43]. Kinematic and EMG data were normalized in the “perturbation” phase to 101 data points starting at the start of the perturbation until the end of the perturbation. The data were also normalized to 101 data points during the “response” phase starting at the end of the perturbation until one second afterwards (Fig 2). Root mean square values were calculated during both the perturbation and response phase for the normalized vastus lateralis (VL), vastus medialis (VM), hamstrings lateralis (HL) and hamstrings medialis (HM) activity.

Each condition consisted of five perturbation trials and all trials were averaged so all the reported variables are means of the five included perturbation trials. Stepping responses were also monitored when balance on the dominant leg could not be maintained during the five second time period after the perturbation (i.e. the participant took a step to prevent falling). Participants used a visual analogue scale (VAS) to indicate the subjective difficulty level in controlling the different external perturbations with zero indicating the perturbation was very easy to control and ten indicating very hard to control. Peak knee flexion and knee abduction angles were calculated. The range of motion of the centre of mass displacement (COM) in medio-lateral and anterior-posterior direction was calculated for both the “perturbation” and the “response” phase. The medio-lateral and anterior-posterior COM were corrected for the absolute movement of the platform in the specific directions. If the medio-lateral COM moved 0.05 m in lateral direction as a response to a lateral single-planar perturbation of 0.03 m, the medio-lateral COM was corrected for the translation of the platform in this specific lateral direction resulting in a corrected medio-lateral COM of 0.02 m.

### Statistical analysis

The average of five trials was calculated for each participant and for each condition. All included variables were normally distributed (Shapiro-Wilk: p > 0.05). Differences within MPP and SPP conditions and between MPP and SPP conditions were investigated for each variable (anterior-posterior and medio-lateral COM, peak knee abduction angle, peak knee flexion angle, VL, VM, HL and HM activity) with one-way analysis of variance tests. Post-hoc Bonferroni tests were used to investigate differences between specific conditions. The statistical level of significance was set at p < 0.05. All statistical analyses were undertaken in Statistica 12 (Statsoft, OK, USA).

## Results

### Differences in responses to multi-planar and single-planar support perturbations

#### Neuromuscular responses

**Fig 3:**
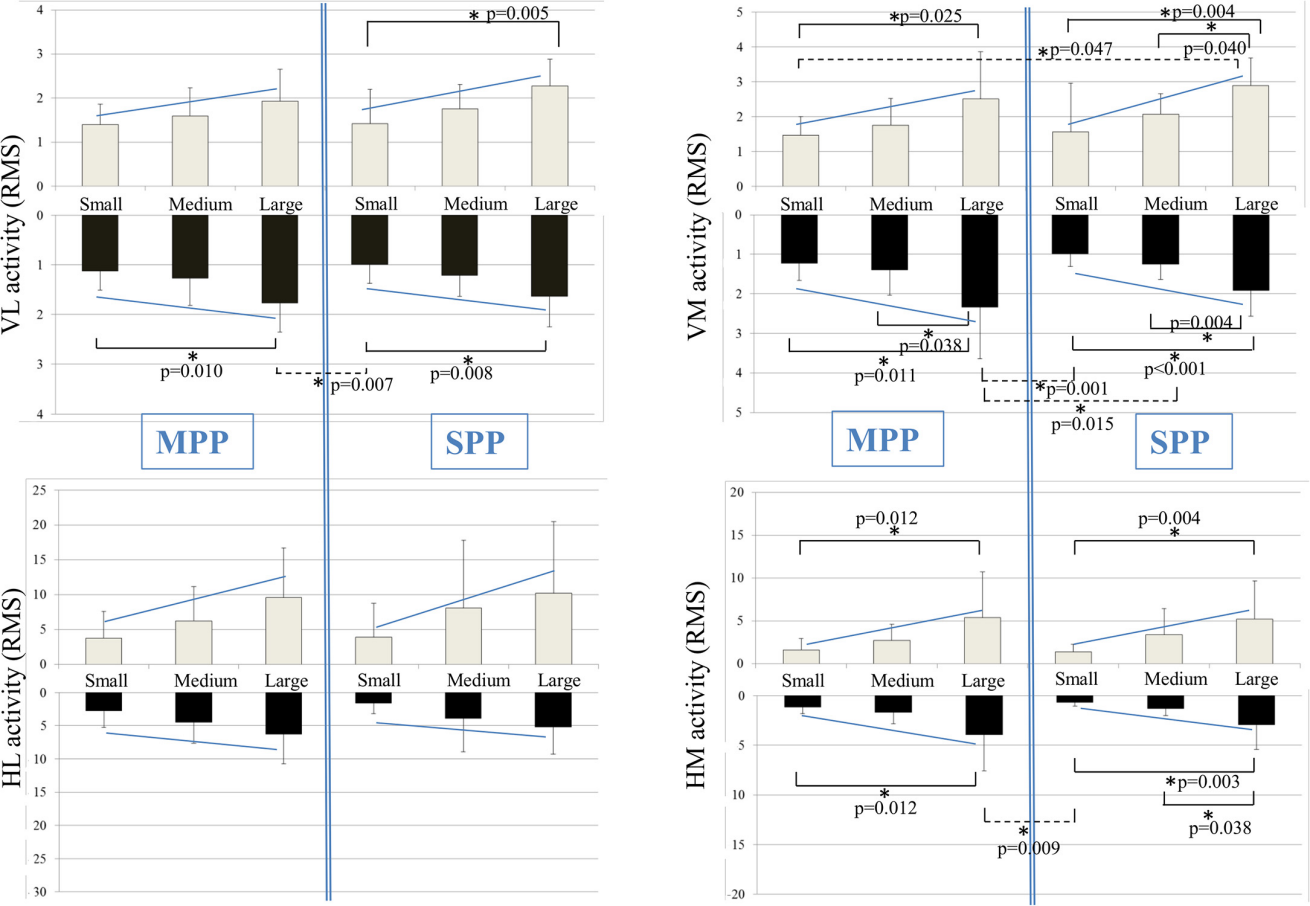
Visualisation of the average muscle activity of the VL, VM, HL and HM in the different conditions. The light colored bars represent the perturbation phase while the dark colored bars represent the response phase. Post-hoc Bonferroni tests revealed significant differences between conditions and are highlighted with an asterisk (p < 0.05).

**Fig 4:**
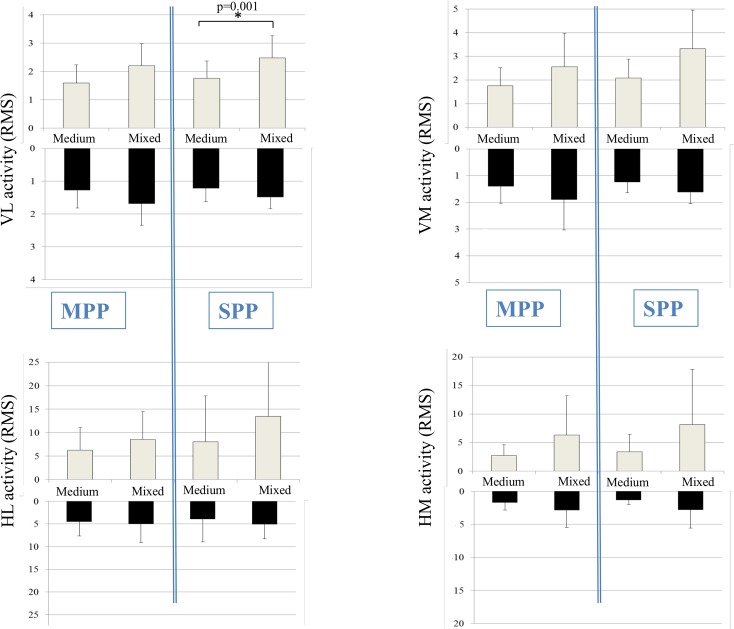
Visualisation of the average muscle activity of the VL, VM, HL and HM in the medium and mixed conditions. The mixed condition consisted of the same amplitude as the medium condition combined with the velocity of the large condition. The light colored bars represent the perturbation phase while the dark colored bars represent the response phase. Post-hoc Bonferroni tests revealed significant differences between conditions and are highlighted with an asterisk (p < 0.05).

No significant differences in muscular activity were found when comparing the MPP-S, MPP-M, MPP-L, MPP-X with their respective equivalent SPP-S, SPP-M, SPP-L, SPP-X during either the perturbation phase or the response phase as can be seen in Fig 3 and Fig 4.

#### COM displacements and kinematics

**Fig 5:**
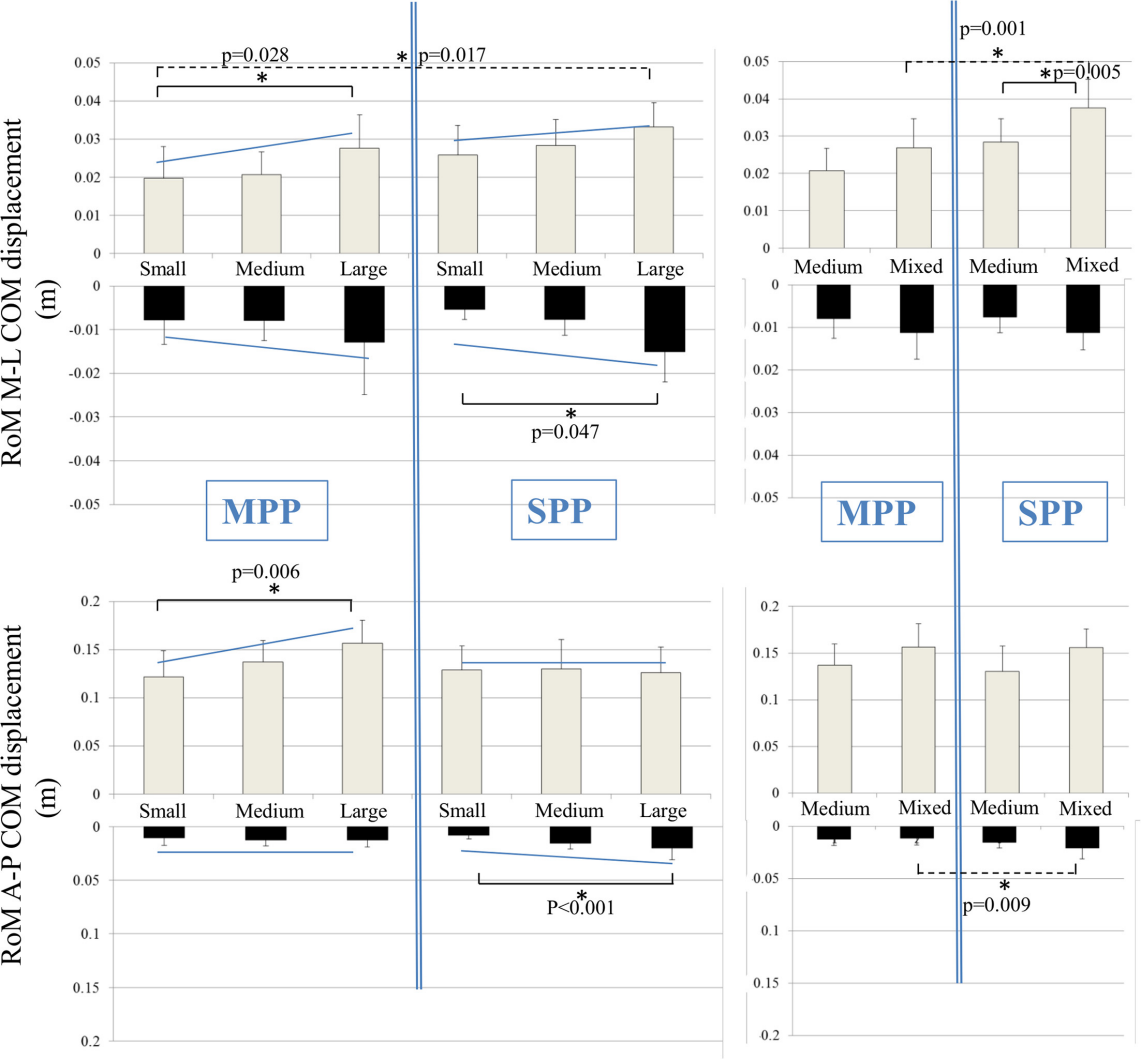
Visualisation of the average medio-lateral and antero-posterior centre of mass (COM) displacement. The light colored bars represent the perturbation phase while the dark colored bars represent the response phase. Post-hoc Bonferroni tests revealed significant differences between conditions and are highlighted with an asterisk (p < 0.05).

Similarly, no significant differences were found in the medio-lateral and anterior-posterior corrected COM displacements between the MPP-S, MPP-M, MPP-L and the equivalent SPP-S, SPP-M, SPP-L. However, the SPP-X resulted in a significantly greater medio-lateral COM displacement compared to MPP-X during the perturbation phase and also in a significantly larger anterior-posterior COM displacement during the response phase as can be seen in Fig 5 (p = 0.001 and p = 0.009, respectively).

No differences were found in peak knee flexion and peak knee abduction angles between the MPP and SPP conditions of similar size (knee flexion angle, MPP-S: 20.87° ± 10.42° vs. SPP-S: 21.61° ± 10.79°; knee abduction angle, MPP-S: 2.50° ± 3.61° vs. SPP-S: 2.48° ± 4.39°).

### Stepping responses and difficulty level (VAS-score)

Nine stepping responses were registered in total during all MPP perturbations while 18 stepping responses were registered during all SPP perturbations (Table 2). Additionally, the subjective VAS-score of the different conditions differed also between the MPP and SPP (Table 2) resulting in higher VAS-scores for the SPP-L and SPP-X compared to the MPP-L and MPP-X.

**Table 2:** The amount of stepping responses that were monitored in the different conditions are visualized in this table as well as the average visual analogue scale (VAS) score relating to the difficulty level to control the different external perturbations.

	Small	Medium	Large	Mixed	
**MPP**	**MPP-S**	**MPP-M**	**MPP-L**	**MPP-X**	**Total**
Stepping responses (n)	0	2	5	2	9
VAS-score (mean±SD)	2.31 ± 1.20	3.77 ± 1.58	4.77 ± 1.76	4.15 ± 1.61	/
**SPP**	**SPP-S**	**SPP-M**	**SPP-L**	**SPP-X**	**Total**
Stepping responses (n)	0	2	10	6	18
VAS-score (mean±SD)	1.69 ± 0.82	3.38 ± 1.00	5.08 ± 1.33	5.15 ± 1.46	/

### Differences within MPP and within SPP conditions

#### Neuromuscular responses

In general, significantly greater VL, VM and HM activity were found in the MPP-L and SPP-L compared to the respective MPP-S and SPP-S during both perturbation and response phases (Fig 3). While no significant differences were found in HL activity between the large and small MPP / SPP, a clear trend showed that conditions that were characterized by greater amplitude, velocity and acceleration resulted in a greater muscular activity pattern in general during both the perturbation and response phase (Fig 3). No significant differences in muscular activity were found between mixed and medium conditions, which are characterized by similar amplitude and different velocity, with the exception of VL activity during the SPP-M and SPP-X (p = 0.034) (Fig 4).

#### COM displacements and kinematics

Similarly, significantly greater medio-lateral and anterior-posterior COM displacements were found in the MPP-L compared to the MPP-S during the perturbation phase (p = 0.028 and p = 0.006, respectively) (Fig 5). The SPP-L showed significantly greater medio-lateral and anterior-posterior COM displacements compared to the SPP-S during the response phase (p = 0.047 and p < 0.001, respectively). In addition, no differences in anterior-posterior COM displacements were found between the SPP-S, SPP-M and SPP-L during the perturbation phase (Fig 5). No differences were found in peak knee flexion and peak knee abduction angles within the MPP and SPP conditions during both the perturbation and response phase (knee flexion angle, MPP-S: 20.87° ± 10.42° vs. MPP-L: 24.27° ± 10.67°; knee abduction angle, MPP-S: 2.50° ± 3.61° vs. MPP-L: 3.60° ± 3.86°).

#### Stepping responses and difficulty level (VAS-score)

The differences in recorded stepping responses and in subjective VAS-score differed within the different MPP/ SPP conditions (Table 2).

## Discussion

The purpose of this study was to compare neuromuscular responses of the quadriceps and hamstring muscles to different types of unexpected multi-planar perturbations (MPP) and different types of unexpected single-planar perturbations (SPP). The results of this study showed significantly greater activity amplitudes of VL, VM and HM during both the perturbation phase and the response phase in conditions that were characterized by greater amplitude, velocity and peak acceleration. Nevertheless, no differences in neuromuscular activity, peak knee flexion and peak knee abduction angles were found comparing the small, medium and large conditions in the MPP versus their equivalents in the SPP.

The increase in neuromuscular response during more challenging conditions confirms previous studies by Horak et al. [28] and Szturm et al. [29]. They also found that the magnitude of muscle activity was scaled towards the amplitude, velocity and acceleration of the platform perturbations. This resulted in higher muscle activity levels in more challenging perturbation conditions. In contrast, recent work of Chen et al. [44] found no effect of a platform’s amplitude and velocity on the magnitude of muscle activation although it did significantly affect the joint movements and the COM displacements. Mixed conditions were also included in the present study. These conditions were characterized by the same amplitude as the medium conditions in combination with the velocity of the large condition. Current results showed that a greater velocity of the platform resulted in higher muscle activity levels while the platform amplitude did not differ between the medium and mixed conditions. This indicates that platform velocity is a crucial variable which induces a greater neuromuscular response.

No differences were found in muscle activity between the more (or less) challenging MPP (MPP-L) versus their equivalent SPP (SPP-L) suggesting that muscular activity was not affected differentially by the multi-planar nature of the MPP in comparison to the SPP taking into account that the velocities and peak accelerations were comparable between MPP and SPP. MPP were characterized by a posterior and lateral translation and additional rotations around the antero-posterior and around the vertical axes while the SPP were characterized by only a lateral translation. Both MPP and SPP induced knee abduction in the participants performing this stepping task, however the additional rotational and translational components in the MPP appear not to influence the muscle activity levels of the quadriceps and hamstrings muscles nor the peak knee abduction and peak knee flexion angles during the MPP.

Whilst it was expected that MPP have provided more stratifying results, it may be that combinations of additional rotations and additional posteriorly orientated translation only, which had little effect on the kinematics and kinetics, were insufficient to tease out differences compared to SPP. It is important to mention that we only included the knee joint kinematics in the sagittal and frontal planes. However, evidence showed the human body acts as a linked-segment model [44]. So, it might be possible that the MPP induced differences in the kinematics of other segments of the human body, i.e. ankle, hip, trunk, etc. Nevertheless, based on these findings we can conclude that the MPP do not elicit different neuromuscular or kinematic responses compared to SPP. The stepping height and/or stepping distance may also influence the lower limb kinematics and neuromuscular control during such a dynamic stepping task. Stepping from a higher box might result in greater neuromuscular response compared to stepping from a smaller box. In the current study, a box of 0.1 m has been used and this might be too easy or insufficiently challenging to examine the neuromuscular control. Therefore, future studies should investigate if a different intensity (stepping height) or stepping distance might elicit neuromuscular or kinematic differences between MPP versus SPP as the current study only investigated one defined stepping height and stepping distance.

The medio-lateral and anterior-posterior COM displacements, were significantly greater in the more challenging MPP and SPP compared to the less challenging MPP and SPP during the perturbation phase in the MPP and during the response phase in the SPP. Remarkably, the anterior-posterior COM displacement during the perturbation phase remained stable during the different SPP conditions. This indicates that amplitude or velocity did not influence the anterior-posterior COM displacements during single-planar perturbations. Additionally, a significantly greater medio-lateral and antero-posterior COM displacement was found in the SPP-X compared to the MPP-X during the perturbation and response phase, respectively. This important difference, suggesting a larger overall postural response to the single-planar perturbations, might be due to a slightly greater peak acceleration in the SPP-X compared to the MPP-X as suggested earlier by Szturm et al. [29]. Current results were in line with earlier findings of Chen et al. who demonstrated that translational perturbations induce more horizontal COM displacements than rotational perturbations [27].

Our purpose was to address the importance of the implementation of using an unexpected perturbation to perturb the neuromuscular homeostasis of an athlete without compromising the ability to perform the screening task. Therefore, it was crucial that the task was not too challenging for participants avoiding that they would be unable to control the perturbation without a stepping response. Eighteen stepping responses were identified in the SPP compared to nine stepping responses in the MPP (twice the number of stepping responses in the SPP) indicating that the overall neuromuscular control was more challenged during the SPP conditions. This is confirmed by the greater anterior-posterior and medio-lateral COM displacements in the SPP-X versus MPP-X. In addition, higher subjective VAS-scores were found in the more challenging SPP compared to the more challenging MPP confirming the greater overall COM displacements in the more challenging SPP. Nevertheless, our purpose was not to assess the overall postural control of the participants.

Caution must be applied when interpreting the findings of this study as some methodological limitations have to be taken into account. It is important to highlight that we have calculated discrete root mean square values during the perturbation itself (perturbation phase) and also during a specified period starting at the end of the perturbation until one second afterwards (response phase). Examination of EMG activities over time [45] may provide additional insight into neuromuscular control strategies. Furthermore onset determinations could provide interesting information regarding the neuromuscular co-activation patterns of the quadriceps and hamstrings. Additionally, as the human body acts as a linked-segment model [46], it might be relevant to include hip, trunk and ankle kinematics and kinetics in future work. To date, limited studies have investigated the use of external support perturbations in the domain of ACL injury prevention [21]. Therefore, we used relatively small perturbations at low velocities and accelerations to maximize safety throughout the test protocol. In addition, future studies should implement the analysis of forces and moments that are acting on the human body during external perturbations as this present study only focused on the neuromuscular activity levels and the kinematics. Finally, the rationale for the development of multi-planar unexpected perturbations in the area of ACL injury prevention was to screen athletes dynamically, in an unexpected way targeting the strong link with real sports situations. Despite the fact that clear evidence showed that ACL injuries mostly occur with the foot firmly positioned on the ground [25], previous evidence showed that unexpected support perturbations might challenge the neuromuscular control as the neuromuscular control is challenged during real sports situation [47]. Additionally, it is important to take into account that there might be a potential lack of power in this study that may have resulted in a Beta error. Therefore, future research is needed to tackle the unexpected perturbation paradigm.

To conclude, the preliminary study on a small sample of healthy control subjects showed no significant differences in muscular activity and in knee joint kinematics between multi-planar perturbations compared to single-planar perturbations. Interestingly, while the muscle activity levels of the quadriceps and hamstrings were similar during the multi- versus their equivalent single-planar perturbations, the overall neuromuscular control appeared less challenged during the multi-planar perturbations. As this study only included few neuromuscular parameters, future work should investigate if differences in co-activation patterns of lower extremity muscles, in lower limb kinetics, and in ground-reaction forces can be found when comparing MPP and SPP. Furthermore, stepping height, stepping distance and perturbation types and magnitudes might also result in a different neuromuscular control when comparing MPP and SPP. Therefore, future research should assess if a higher stepping height or a greater stepping distance do elicit neuromuscular differences when comparing MPP versus SPP.
